# Controlled Synthesis of Polyions of Heavy Main-Group Elements in Ionic Liquids

**DOI:** 10.3390/ijms17091452

**Published:** 2016-09-01

**Authors:** Matthias F. Groh, Alexander Wolff, Matthias A. Grasser, Michael Ruck

**Affiliations:** 1 Department of Chemistry and Food Chemistry, Technische Universität Dresden, 01062 Dresden, Germany; matthias.groh@chemie.tu-dresden.de (M.F.G.); alexander.wolff@chemie.tu-dresden.de (A.W.); matthias.grasser@chemie.tu-dresden.de (M.A.G.); 2 Max Planck Institute for Chemical Physics of Solids, Nöthnitzer Strasse 40, 01187 Dresden, Germany

**Keywords:** amines, ionic liquids, ionothermal, main-group elements, modifications, polyanions, polycations, starting materials, synthesis parameters, vapor pressure

## Abstract

Ionic liquids (ILs) have been proven to be valuable reaction media for the synthesis of inorganic materials among an abundance of other applications in different fields of chemistry. Up to now, the syntheses have remained mostly “black boxes”; and researchers have to resort to trial-and-error in order to establish a new synthetic route to a specific compound. This review comprises decisive reaction parameters and techniques for the directed synthesis of polyions of heavy main-group elements (fourth period and beyond) in ILs. Several families of compounds are presented ranging from polyhalides over carbonyl complexes and selenidostannates to homo and heteropolycations.

## 1. Introduction

Ionic liquids (ILs)—Often defined as salts with melting points below 100 °C—have actually been known for quite a long time. In the decades after the description of the first representative ethylammonium nitrate by Paul Walden in 1914 [1], this valuable family of compounds fell into oblivion. Despite pioneering works [2], there had only been approximately 20 articles per year on ILs until 1995 [3]. Beginning with the late 1990s, the annual number of publications rose tremendously to about 9200 in the year 2015, according to “Web of Science”, indicating the huge interest of different research communities.

Usually, ILs are constituted from bulky organic cations and (often) polyatomic anions, which can be selected in order to tune the properties of the IL. Typical cations range from simple quaternary ammonium or phosphonium ions over substituted imidazolium or pyridinium rings to more complex cations, such as the so-called TAAILs (Tunable Aryl Alkyl Ionic Liquids) [4]. A similar variety can be found among the anions including, for example, simple halides, complex organic anions, or halogenidometalates [5]. Among others, the latter can be utilized to introduce additional physical properties like magnetic moments [6].

Owing to the distinctive physicochemical properties of ILs (wide liquidus range, high redox and thermal stability, negligible vapor pressure, and tunable polarity) and their advantages over organic solvents, classic melts, or solid state reactions, their applications include separation techniques [7,8,9], lubrication [10], electrodeposition [11], acting as electrolytes in photovoltaic devices (e.g., solar cells) [12,13,14], catalysis for clean technology [15,16,17,18], polymerization processes [19], crystal engineering of a wide range of inorganic substances [20,21,22,23,24,25], and syntheses of new inorganic materials in general [23,26,27,28,29,30,31,32,33,34,35,36,37,38,39,40,41,42,43,44,45,46,47,48,49,50,51]. Recently, several comprehensive reviews on syntheses of inorganic compounds in ILs have been published by Taubert [52], Feldmann [28], Dehnen [51], Janiak [42,53], Scrosati and Passerini [48], Morris [54], Mudring [55], Prechtl [56], Zhu [57], Dai [58], and Ruck [23,27,59] among others.

Despite the abundance of inorganic compounds yielded by IL approaches, the syntheses remain a “black box” in several cases. Not only are the mechanisms of product formation barely examined but the overall role of the IL might also be vague. The latter can range from being a mere lubricant for solid state reactions via acting as solvent for (a part of) the starting materials to crucial directive properties leading to tunable products. In addition, the interplay of ILs with additives or directing agents has to be investigated further. Therefore, either in situ reaction monitoring [60,61,62] or comparisons of several syntheses can elucidate the influence of ILs. The present review article aims to highlight a variety of reaction parameters and techniques for the directed synthesis of polyions of heavy main-group elements (fourth period and beyond) in ILs (cation and anion abbreviations in Table 1) in order to provide researchers with an insight into promising synthetic approaches. We will summarize or deduce crucial reaction parameters and techniques and—in several cases—demonstrate the benefit of utilizing an IL in comparison to classic synthetic routes. The review is subdivided into sections on polyanions and polycations, and further into subsections about specific subgroups with their decisive reaction parameters.

## 2. Polyanions

Several polyanions, among them also polyoxometalates [63], have been synthesized in ILs. Herein, we focus on heavy polyhalides, carbonyl clusters, and selenidostannates. Owing to the abundance of synthesized compounds, crucial reaction parameters as well as the influence or benefit, respectively, of the ILs can be deduced.

### 2.1. Starting Materials with High Vapor Pressure

The ability of ILs to decrease the vapor pressure of delicate volatile compounds and, simultaneously, to provide access to dissolved species has been utilized for the synthesis of several heavy main-group element polyanions. In order to demonstrate the potential of this method, we discuss the utilization of halogens and metal carbonyls in IL-based syntheses.

The interplay between ILs and gases has been studied intensively for many years. Some gases, for example CO_2_ and SO_2_, proved to feature a remarkable solubility in some ILs [16,64,65,66]. The interplay between gaseous species and ILs can be manifold [64]. For CO_2_, interactions with the anions of ILs have been observed [67], and the gases PH_3_ and BF_3_ are dissolved via chemical complexation [68].

#### 2.1.1. Polyhalides

**Figure 1:**
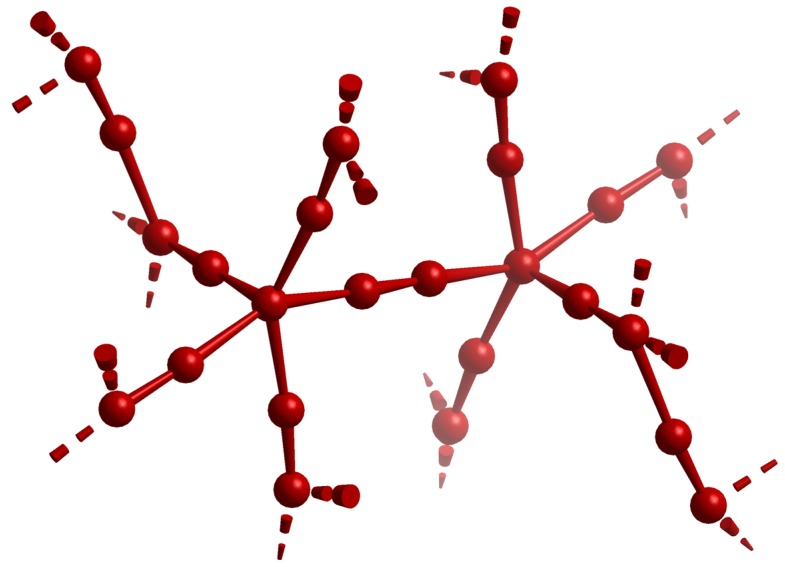
Visualization of the anion [Br_24_]^2−^ according to [71]. Br–Br distances with d ≤ 320 are drawn as solid lines to emphasize the [Br_24_]^2−^ unit. Additional Br–Br distances up to 370 pm (i.e., twice the van der Waals distance [71]; dashed broken-off bonds) indicate the network character.

Intriguing examples for the utilization of halogens are the polybromides synthesized in ILs [43,69,70,71,72]. While polyiodides were known with up to 29 iodine atoms [73], the size of the corresponding polybromides was limited to a maximum of 10 atoms [74,75] before new approaches including ILs (Feldmann group) were developed [76]. This disparity is most likely due to the vapor pressure of bromine (10 kPa at 2.5 °C, b.p.: 58.8 °C) [77], which is considerably higher than for iodine (10 kPa at 108 °C, b.p.: 184.4 °C) [77]. Moreover, the reactivity of the lighter halogens necessitates a chemically stable environment, which can be provided by ILs due to their high redox stability [78]. The use of ILs lead to the isolation of several new polybromides: [P(Ph_3_)Br][Br_7_] [69] ([P(Ph_3_)Br] = triphenylphosphine bromide), [P(Bz)(Ph)_3_]_2_[Br_8_] [69], [HMIm][Br_9_] [70], [N(n-Bu)_3_Me]_2_[Br_20_] [69], [C_4_MPyr]_2_[Br_20_] [43,69], and [P_4444_]_2_[Br_24_] (Figure 1) [71]. The anion of [C_4_MPyr]_2_[Br_20_] was regarded as the bromine-richest species (aside from the element itself) at its discovery by Wolff et al. [43]. This record has been excelled by the synthesis of [P_4444_]_2_[Br_24_] and its [Br_24_]^2−^ anion by Easton et al. [71].

All IL-based syntheses of polybromides rely on one strategy: Bromide anions dissolved in an IL act as electron donors to bromine molecules. The characteristic red-brown vapor of bromine is missing above the IL at moderate temperatures [69] indicating the high solubility in and strong interactions with the IL. The same effect was also observed for iodine in the course of the synthesis of phosphorus iodides [60]. For convenient isolation of the solid product, the reaction mixture has to be liquid at room temperature or, in some cases, even below, which can be achieved by different means: either a pure room-temperature ionic liquid (RTIL), for example [HMIm][Br], is used [70] or auxiliaries, like (2-bromophenyl)diphenylphosphine, are added to the RTIL [N(n-Bu)_3_Me][NTf_2_] [69]. Another possibility is the admixture of a second IL to form eutectic mixtures, for example [P_4444_][Br]/[P_44414_][NTf_2_] [69] or [C_10_MPyr][Br]/[C_4_MPyr][OTf] [43,69].

Interestingly, only two compounds, [P(Ph_3_)Br][Br_7_] and [P(Bz)(Ph)_3_]_2_[Br_8_], were obtained by cooling the heated reaction mixture to room temperature [69]. To achieve crystallization of all other compounds (with larger anions), deeper cooling was necessary. In the case of [C_4_MPyr]_2_[Br_20_], the liquid was initially cooled to −15 °C, which lead to the crystallization of the target compound and the IL itself. By reheating to +5 °C, only crystals of the polybromide remained [43]. It seems worth noting that crystallization is only achieved if the cations are not too bulky. In the case of [P(Ph_3_)Br][Br_7_], adding triphenylphosphine to the eutectic mixture of [C_10_MPyr][Br] and [C_4_MPyr][OTf] initiated crystallization [69]. In further investigations, the structure of the IL cation was identified to be the most important parameter with respect to the bromine content of the product. Therefore, further optimizing the cation-bromine interactions might lead to polybromides with even higher bromine contents [72].

#### 2.1.2. Utilization of Metal Carbonyls

Handling metal carbonyls can be cumbersome due to their high vapor pressure and toxicity. Thus, introducing a reaction medium for saver storage and handling of carbonyls would be highly beneficial. First investigations by Brown et al. on the chemistry of carbonyl compounds in ILs focused on the formation of [BMIm][Co(CO)_4_] from the reaction of [BMIm]Cl with Na[Co(CO)_4_] in propanone. After removing the organic solvent, the resulting blue-colored IL [BMIm][Co(CO)_4_] did not show any mass loss, even when stored under vacuum for 48 h, indicating the high stability of this liquid salt and its ability to provide “dissolved” carbon monoxide [79].

The first IL-based synthesis of carbonyl clusters of heavy main group elements were reported by the Feldmann group (Table 2). Mn_2_(CO)_10_ or Fe(CO)_5_ were reacted with metalloid iodides in different ILs at 130 °C. Similar to the aforementioned case of polybromides, the reaction temperature is remarkably high considering the vapor pressure and boiling point of e.g., Fe(CO)_5_ (10 kPa at 44 °C, b.p.: 103 °C) [77].

Some of the CO ligands remain bonded to the transition metal atom in the product. It is noteworthy that gaseous CO itself is just sparingly soluble in ILs [64], which matches the observation of an excess pressure of the reaction by-product CO while opening the sealed ampules [80]. Therefore, metal carbonyl fragments from the starting material exist under the chosen reaction conditions and are thus dissolved and available for further reactions.

**Figure 2:**
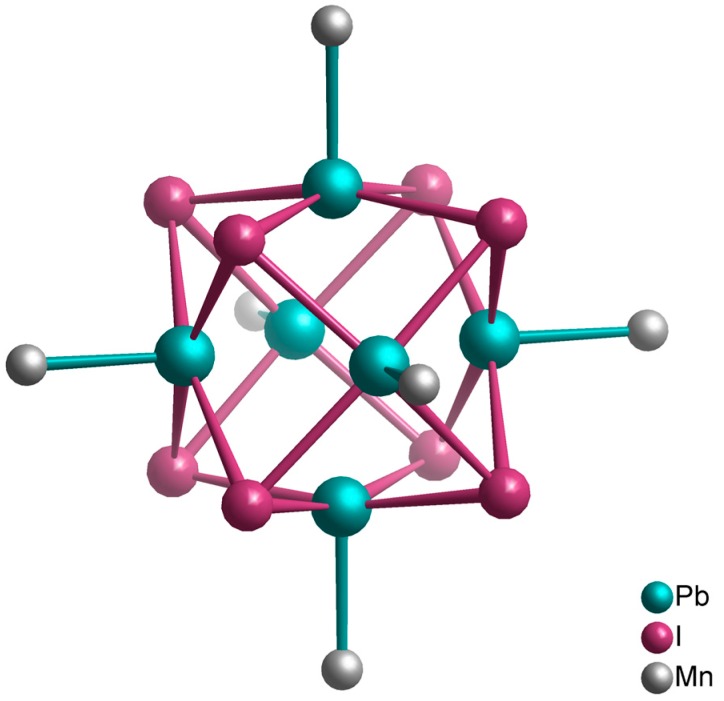
Visualization of the anion [(Pb_6_I_8_){Mn(CO)_5_}_6_]^2−^ in [BMIm]_2_[(Pb_6_I_8_){Mn(CO)_5_}_6_] [81]. The carbonyl ligands at the manganese atoms are omitted for clarity.

The stability of the obtained compounds varies. On the one hand, [BMIm]_2_[(Pb_6_I_8_){Mn(CO)_5_}_6_] (Figure 2) releases CO at room temperature if isolated from the IL [81]. Crystals of [BMIm]_2_[{Fe(CO)_3_}_4_Sn_6_I_10_] and [BMIm]_6_[S][{Fe(CO)_3_}_4_Sn_6_I_10_]_2_ release CO upon squeezing [83]. [BMIm][(Te_2_)_3_{Mn(CO)_3_}_2_{Mn(CO)_4_}_3_], on the other hand, is thermally stable up to 380 °C [80]. According to investigations by Feldmann et al., the sidechain length at the IL cation as well as the nature of the IL anions have no influence on the product formed (Table 2) [82,83].

In conclusion, the utilization of ILs allows safe handling of compounds with high vapor pressure like bromine or metal carbonyls at reaction temperatures above their boiling point due to their exceptional solubility in chosen ILs. Furthermore, dissolved species such as metal carbonyl fragments are available for reactions with, e.g., compounds of heavy main-group elements. In the case of the polybromides, the structure of the IL cation proved to be the dominant parameter for product-selective synthesis.

### 2.2. Amine-Assisted Syntheses of Selenidostannates

Structure-directing properties of ILs for the synthesis of inorganic materials are known since the initial studies of the Morris group [84,85,86]. They also noticed that the addition of small amounts of molecular solvents, e.g., H_2_O, has a strong effect on the formation of the products if combined with ILs [87]. This feature was further explored during the ionothermal synthesis of molecular sieves with auxiliary amines [61,62,88,89]. The groups of Dehnen and Huang reported similar findings for clusters of heavy main-group elements. They noticed that the addition of amines had strong impact on the phase formation of selenidostannates synthesized from the elements or prereacted species in ILs [51,90,91,92,93,94,95,96,97,98]. More than 25 different selenidostannates were synthesized following this approach, which does not only illustrate the diversity of this class of compounds but also demonstrates the capability of the method. More details are given in a recent review about the synthesis and structure of selenidostannates by Dehnen et al. [51].

#### 2.2.1. Promoting Phase Formation

**Figure 3:**
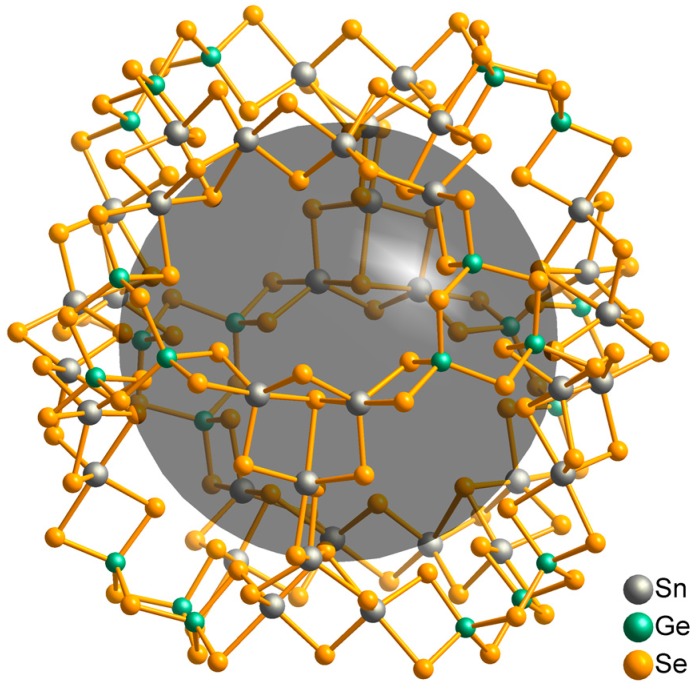
Visualization of the “zeoball” anion [Sn_36_Ge_24_Se_132_]^24−^ in [BMMIm]_24_[Sn_36_Ge_24_Se_132_] [91]. The disorder has been omitted for clarity. The dark sphere with a diameter of 1.54 nm indicates the inner void of the anion.

There are many examples in which products have exclusively been obtained in the presence of an amine including the famous “zeoball”-type selenidostannates [BMMIm]_24_[Sn_36_Ge_24_Se_132_] (ZBT-1) and [BMIm]_24_[Sn_32.5_Ge_27.5_Se_132_] (ZBT-2), which feature the largest known discrete polyanion of main-group elements (Figure 3) [91]. Both compounds were synthesized from [K_4_(H_2_O)_3_][Ge_4_Se_10_] and SnCl_4_∙5H_2_O in tetrafluoridoborate ILs in the presence of DMMP (DMMP = 2,6-dimethylmorpholine). Noteworthy, only uncharacterized Ge/Se-containing powder precipitated if the reactions were conducted in the absence of the amine, whereas an excess of it led to microcrystalline SnSe_2_. In another reaction, SnCl_4_∙5H_2_O was replaced with [K_4_(H_2_O)_4_][SnSe_4_], which contains a preformed binary unit of tin and selenium atoms. This led to the formation of ZBT-2 in the absence of DMMP. This strongly evidences that the amine is involved in the formation of initial binary (or higher) species of tin and selenium. In further experiments, [K_4_(H_2_O)_3_][Ge_4_Se_10_] and SnCl_4_∙5H_2_O were reacted with en (en = ethylenediamine) instead of DMMP, which led to the formation of ZBT-1 at a remarkably lower amine to IL ratio, probably due to the higher base strength of en [92].

#### 2.2.2. Phase Selectivity

Following the above-mentioned syntheses, more detailed experiments have been performed in the same system by Lin et al. aiming for a deeper understanding of the role of the amine. Different reactions in [BMMIm][BF_4_] were performed under invariant conditions and only the amount of the added amine was changed (Table 3). According to these experiments, the basicity of the amine has strong influence on the phase formation [92]. With increasing basicity, the overall tendency of the Ge/Se and Sn/Se subunits to aggregate is decreased and the incorporation of tin into the anionic substructure is favored (Table 3). In particular, products could be precisely targeted by switching the amine from DMMP to en. Adding larger amounts of en led exclusively to the formation of a 3D network, whereas the layered (2D) and the cluster (0D) compounds are formed exclusively at lower concentrations [92].

In the case of the synthesis of binary selenidostannates from elemental Sn and Se by Li et al. (Table 4), 3D-[PMMIm]_4_[Sn_9_Se_20.93_] was formed consisting of [SnSe_4_] and [SnSe_3_(Se_2_)_0.9_Se_0.1_] tetrahedra, which are connecting [Se_3_Se_4_] semicubes. In contrast to the work of the Dehnen group, increasing the amount of amine led to the formation of 2D-[PMMIm]_2_[Sn_3_Se_7_] [90,94]. It becomes evident that tuning only the amount of amine does not necessarily determine the dimensionality of the crystallized anion. Increased addition of amines led to two-dimensional anions in the case of binary anions and to 3D-networks for ternary ones (Table 3). This is most likely because other factors, e.g., temperature or the shape of the cation, also play an important role [90,93,94]. Therefore, if an undesired dimensionality results from a synthesis with auxiliary amines, increasing as well as decreasing of the amine content should be considered.

#### 2.2.3. Crystal Growth

As mentioned above, it was possible to synthesize the “zeoball” compound ZBT-2 without addition of amines. However, it was noted that crystal quality and yield were poor compared to the amine-assisted synthesis [91]. Therefore, the amines apparently play a role during crystal growth.

**Figure 4:**
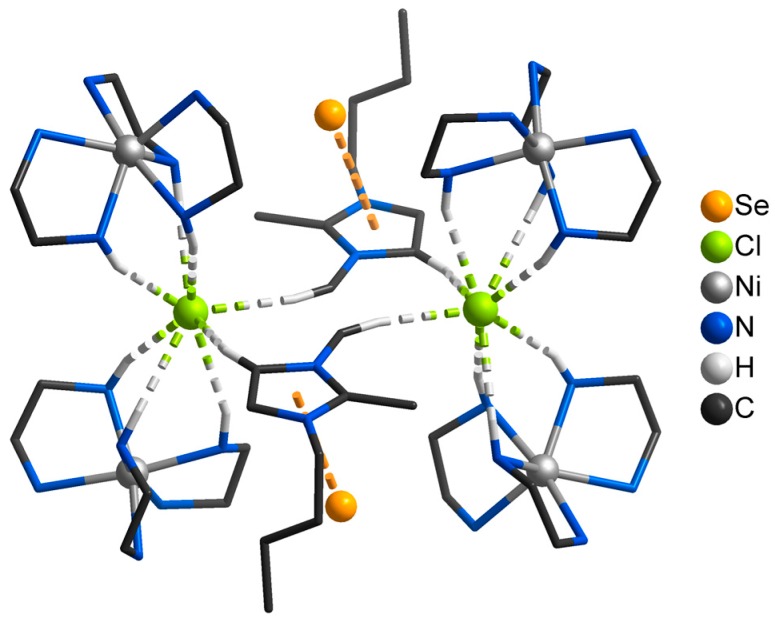
Visualization of the metal-amine complexes (MAC) in [BMMIm]_3_[Ni(en)_3_]_2_[Sn_9_Se_21_]Cl [98]. The disorder and the anion except for one selenium atom each is omitted for clarity. Hydrogen bonds are pictured as green-grey dashed lines and anticipated anion-π interactions as orange dashed lines. Hydrogen atoms are only depicted if participating in bonds toward chlorine atoms. The figure was developed according to [98].

This phenomenon has also been observed in NMR experiments during the synthesis of molecular sieves [61]. In their experiments, Xu et al. found that imidazolium cations form hydrogen bonds with the amine molecules during crystallization. These intermediates were identified as structure directing agents for the forming solid. The imidazolium cations themselves are pore-filling agents during crystal growth [61,62]. The influence of hydrogen bonding in the course of crystallization was also observed for the transformation of 2D-[BMMIm]_16_[Sn_24_Se_56_] into 1D-[BMMIm]_4_[Sn_6_Se_14_] or 1D-[BMMIm]_3_[DMMPH][Sn_6_Se_14_]. Heating 2D-[BMMIm]_16_[Sn_24_Se_56_] to 150 °C led to the partial transformation into 1D-[BMMIm]_4_[Sn_6_Se_14_], whereas 1D-[BMMIm]_3_[DMMPH][Sn_6_Se_14_] was obtained under the same conditions by adding DMMP. Interestingly, the yield of 1D-[BMMIm]_4_[Sn_6_Se_14_] is low even after long reaction times. The transformation into 1D-[BMMIm]_3_[DMMPH][Sn_6_Se_14_], however, is almost quantitative after two days. Lin et al. supposed that the protonation of DMMP and the resulting DMMPH cation is advantageous for the formation of the 1D structure, which is otherwise seemingly disfavored. Additionally, the transformation can be reversed by reacting any of the 1D-compounds with en. It was pointed out that in this case, en might play an important role as auxiliary agent that forms intermediate selenium-hydrogen bonds [93]. Indeed, hydrogen bridges between en and selenium were later recognized in selenenidostannates with metal-amine complexes (MACs) acting as cations [95,96,97,98]. For example, in (BMMIm)_3_[Ni(en)_3_]_2_[Sn_9_Se_21_]Cl, hydrogen bonds have been found between en and the selenium atoms of the [∞2Sn_3_Se_7_]^2−^ layers. However, this compound also demonstrates the complexity and diversity of different interactions between the MAC, the imidazolium cation, the chloride anion, and the selenium atoms of the selenidostannate layer (Figure 4) [98].

In conclusion, the combination of amines and ILs enabled the synthesis of a variety of selenidostannates. The introduction of auxiliary amines provides several additional reaction parameters. The amount, structure, and basicity of the amine can influence the dimensionality of the polyanions as well as their crystallization. The reaction mechanism includes formation of hydrogen bonds between the amines and dissolved starting materials as well as the cations of the ILs. Thus, utilization of amines or comparable auxiliaries could be beneficial for many other syntheses.

## 3. Polycations

### 3.1. Homopolycations—Adjustments via Redox Potential and Starting Materials

While polyanions are generally synthesized in (Lewis-)basic media, polycations of heavy main group elements are usually obtained from (Lewis-)acidic solutions such as oleum or more generally in systems with only weakly coordinating anions or solvent molecules such as Na[AlCl_4_] melts or liquid SO_2_. Weakly coordinating anions (e.g., weak Lewis bases) are necessary since strong Lewis bases destabilize polycations. The introduction of ILs has increased the convenience of polycation syntheses and enabled substitution of toxic substances such as benzene, SO_2_, or AsF_5_ [99]. Commonly used Lewis-acidic ILs are combinations of alkylimidazolium halides with more than equimolar amounts of aluminum or gallium trihalides (*MX*_3_). The excess of trihalides is beneficial for several reasons: Free *MX*_3_ or their condensation products with [*M*X_4_]^−^ anions such as [*M_2_*X_7_]^−^ act as scavengers for halide ions or other Lewis bases. This leads to increased solubility for metalloid halides (e.g., Bi*X*_3_ dissociates into Bi*X*_2_^+^ and *X*^−^) and partly self-drying ILs (if the Lewis base is water) protecting the formed polycations from hydrolysis. In addition, the concentration of the different halogenidometalate species can be tuned by temperature [100], mole fraction of employed *MX*_3_ [5], and by adding additional free *X*^−^ anions [5100]. The latter has been utilized by Ruck et al. to overcome hindered crystallization of products by adding small amounts of NaCl after completion of the reaction to increase the mole fraction of [AlCl_4_]^−^ [23,26,101].

A variety of homoatomic polycations of group 15 or 16 elements has been synthesized in ILs [26,27,31,99,101,102,103,104] and several influencing reaction parameters were deduced.

#### 3.1.1. Bismuth Homopolycations

Bismuth is famous for its ability to form ligand-free homopolyanions and especially homopolycations. Three of the latter could be synthesized in Lewis-acidic ILs: Bi_5_^3+^ [99,104], Bi_8_^2+^ [26], and Bi_9_^5+^ [26,27]. In general, IL-based syntheses of bismuth polycations are advantageous compared to classic syntheses by increasing purity and yield in addition to lowering the reaction temperature [26].

The first synthesis of Bi_5_^3+^ by reacting elemental bismuth with BiCl_3_ (molar ratio 3:1) in [BMIm]Cl·1.3AlCl_3_ at room temperature was established by Ahmed et al. in 2009 [99]. As continuative experiments in our group have shown, the resulting Bi_5_[AlCl_4_]_3_ is strongly favored in this system as long as redox reactions involving bismuth might occur and especially during reactions at elevated temperature and in highly Lewis-acidic ILs. In fact, Bi_5_[AlCl_4_]_3_ can be regarded as an omnipresent (and inconvenient) side product which has to be prevented from crystallization. Reducing BiCl_3_ with transition metals or other moderate reducing agents typically leads to Bi_5_[AlCl_4_]_3_.

In order to suppress Bi_5_[AlCl_4_]_3_ and to obtain other homopolycation compounds, several strategies have proven to be viable. One possibility is the introduction of bromine, which seems to disfavor the crystallization of Bi_5_^3+^: By reacting equimolar amounts of Bi and BiBr_3_ in [BMIm]Cl·2AlCl_3_ at room temperature, pure Bi_6_Br_7_, which includes Bi_9_^5+^ polycations, is accessible. A second approach on targeting the type of crystallized polycations is by adjusting the reducing agent: The Bi_8_^2+^ polycation is accessible as Bi_8_[AlCl_4_]_2_ by reduction of BiCl_3_ with elemental sodium (molar ratio 1:2.8) in [BMIm]Cl·3.6AlCl_3_ at 140 °C with bismuth, Na[AlCl_4_], and Bi_5_[AlCl_4_]_2_ as byproducts. Lowering the reaction temperature to 80 °C and using a less Lewis-acidic IL with 1.3 equivalents of AlCl_3_ leads to Bi_8_[AlCl_4_]_2_ with Na[AlCl_4_] and bismuth as the only by products [26]. Utilizing indium, however, changes the precipitating cation to Bi_9_^5+^. Bi_6_Cl_7_ can be obtained at room temperature by the reduction of BiCl_3_ with indium (molar ratio 3:2) in [BMIm]Cl·2AlCl_3_ [26]. The latter result might also be attributed to the oxidation of indium into (probably) InCl_3_, which should interact with the IL as additional Lewis acid and could tune the reaction not (only) via the redox potential.

Kloo et al. explored the formation of the Bi_5_^3+^ cation by reducing BiCl_3_ with elemental gallium in different ILs with GaCl_3_ as Lewis acid in different ratios [104]. Thereby, they employed [DMIm]Cl, [C_4_MPyr]Cl as well as [P_66614_]^+^ in combination with Cl^−^, [BF_4_]^−^, [PF_6_]^−^ and [NTf_2_]^−^. In all cases, Bi_5_[GaCl_4_]_2_ was obtained. However, they encountered difficulties to isolate single-crystalline products in all reactions [104].

#### 3.1.2. Tellurium Homopolycations

**Figure 5:**
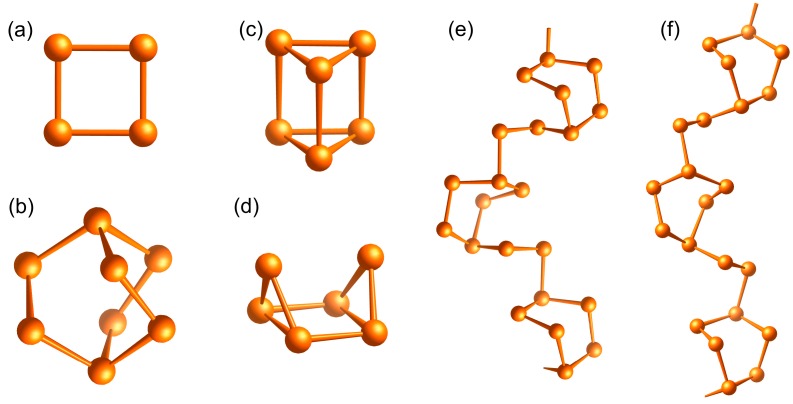
Synopsis of tellurium homopolycations accessible in ILs: (**a**) Te_4_^2+^ e.g., in Te_4_[AlCl_4_]_2_ [101] or “Te_4_^1.78+^” in Te_4_[Bi_0.74_Cl_4_] [31]; (**b**) Te_8_^2+^ in Te_8_[NTf_2_]_2_ [102]; (**c**) Te_6_^4+^ in Te_6_[OTf]_4_ [102]; (**d**) Te_6_^2+^ in Te_6_[WOCl_4_]_2_ [101]; (**e**) [∞1Te_8_]^2+^ in [Te_8_]_2_[Ta_4_O_4_Cl_16_] [103]; and (**f**) [∞1Te_8_]^2+^ in Te_8_[Bi_4_Cl_14_] [26].

Tellurium forms a large variety of polycations. Apart from Te_4_^2+^, which proves a dominance in Lewis-acidic AlCl_3_-containing ILs similar to Bi_5_^3+^, several other tellurium polycations (Figure 5) were accessed in ILs [26,27,31,101,102,103]. Thereby, the utilization of an IL as reaction medium can lead to extraordinary properties of the reaction product as the following example demonstrates: Te_4_[Bi_0.67_Cl_4_] obtained from a gas-phase transport reaction is an conventional semiconductor. However, the closely related Te_4_[Bi_0.74_Cl_4_], obtained from an IL-based low-temperature synthesis, proved to be a one-dimensional metal and type-I superconductor [31].

In the system Te–Bi–Cl, three different ternary compounds (and polycations) can be synthesized under identical conditions in the Lewis-acidic IL [BMIm]Cl·*n*AlCl_3_ (*n* = 1.3–1.5): the aforementioned Te_4_[Bi_0.74_Cl_4_] [31] as well as the closely related semiconductors Te_4_[Bi_6_Cl_20_] [26] and Te_8_[Bi_4_Cl_14_] [26]. The distinctive directing parameter of these room temperature reactions has proven to be the ratio of the starting materials: If elemental tellurium, TeCl_4_, and BiCl_3_ are utilized in a proportion according to the respective composition, each compound is yielded as phase-pure product. In the case of the super-conductor Te_4_[Bi_0.74_Cl_4_], the amount of BiCl_3_ can be increased to 3 equivalents and still the sole crystallization of the desired product does occur.

Omitting any bismuth-containing starting materials leads to the formation of pure Te_4_[AlCl_4_]_2_ under the same conditions [101]. Te_4_[Al_2_Cl_7_]_2_ has been synthesized during an attempt to access phosphorus-tellurium polycations by reacting equimolar amounts of tellurium, TeI_4_, and red phosphorus in the same IL with *n* = 4.8 at 80 °C [26]. The crystallization of the identical polycation but with [Al_2_Cl_7_]^−^ as anion can be rationalized with the higher content of AlCl_3_ resulting in a virtual absence of dissolved [AlCl_4_]^−^ [5].

Introducing other redox agents and simultaneously forming anions has shown to alter the obtained tellurium homopolycation. Ahmed et al. obtained pure Te_6_[WOCl_4_]_2_ by oxidizing elemental tellurium using WOCl_4_ under otherwise identical conditions compared to the formation of Te_4_[AlCl_4_]_2_ [101]. Similarly, Feldmann et al. obtained [Te_8_]_2_[Ta_4_O_4_Cl_16_] by reacting elemental tellurium, TeCl_4_, TaOCl_3_, and TaCl_5_ in the molar ratio of 7:1:2:1 in [BMIm]Cl [103].

A different approach was chosen by Beck et al.: Combining their incomparable expertise on tellurium polycations with the known suitability of ILs as electrolytes resulted in the novel synthesis of tellurium polycations via anodic oxidation of elemental tellurium in ILs. They obtained Te_4_[CTf_3_]_2_, Te_6_[Otf]_4_, and Te_8_[NTf_2_]_2_ after one to three weeks depending on the chosen IL and voltage [102]. By applying 4 V in the IL [EMIm][OTf], a red-violet anodic solution was obtained which yielded [Te_6_][OTf]_4_ upon layering or washing with liquid SO_2_ at −60 °C. Te_8_[NTf_2_]_2_ was synthesized with a voltage of 6 V at 50 °C in [N(n-Bu)_3_Me][NTf_2_]. The elevated temperature proved to be necessary due to the high viscosity of the chosen IL, which limited the cell current. Slow diffusion of CH_2_Cl_2_ into the IL initiated the crystallization of the product. Finally, in the synthesis of Te_4_[CTf_3_]_2_, [BMIm][CTf_3_] acted as conducting salt in liquid SO_2_ at −60 °C and for voltages of 4–6 V. The precipitation was achieved by slow evaporation of SO_2_.

### 3.2. Antimony-Selenium Heteropolycations—Auxilaries, Temperature, and Influence of Halogens

**Figure 6:**
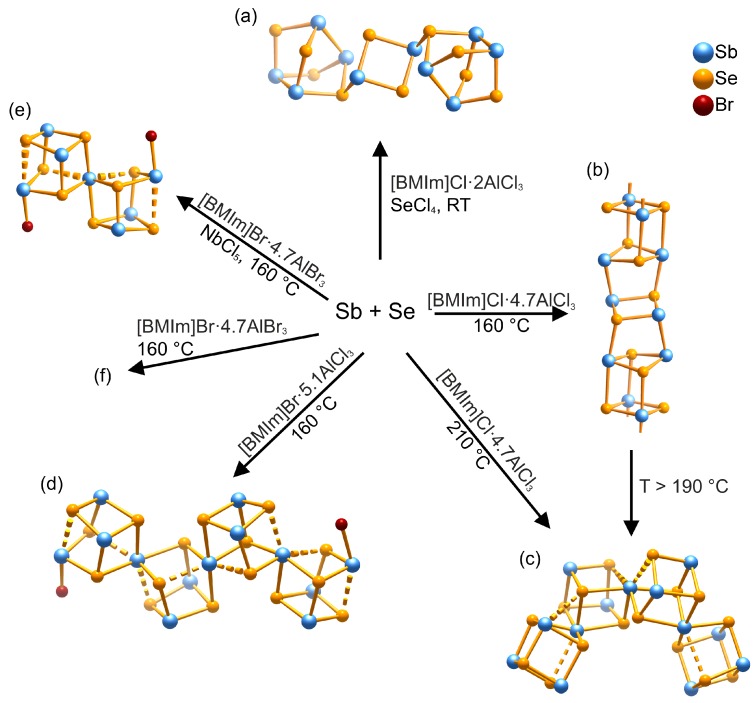
Synopsis of antimony-selenium heteropolycations accessible in ILs and their respective synthetic approach. (**a**) [Sb_10_Se_10_]^2+^ in [Sb_10_Se_10_][AlCl_4_]_2_ [35]; (**b**) [∞1Sb_2_Se_2_]^+^ in [Sb_2_Se_2_][AlCl_4_] [34]; (**c**) [Sb_13_Se_16_]^7+^ in [Sb_13_Se_16_][AlCl_4_]_6_Al_2_Cl_7_ [30,34]; (**d**) [Sb_13_Se_16_Br_2_]^5+^ in [Sb_13_Se_16_Br_2_][Al*X*_4_]_5_ [38]; (**e**) [Sb_7_Se_8_Br_2_]^3+^ in [Sb_7_Se_8_Br_2_][Al*X*_4_]_3_ [38]; and (**f**) [Sb_7_Se_8_Br_2_]^3+^ and [Sb_13_Se_16_Br_2_]^5+^ coexist in [Sb_7_Se_8_Br_2_][Sb_13_Se_16_Br_2_][AlBr_4_]_8_ [38]. Dashed lines represent longer distances that might be interpreted as secondary bonds [30,38].

In recent years, we synthesized several binary or ternary antimony-selenium heteropolycations (Figure 6) in Lewis-acidic ILs [BMIm]*X*·*n*Al*X*_3_ (*X* = Cl, Br; *n* = 1.2–5.2) [30,34,35,38]. Thereby, we deduced two main decisive reaction parameters for the controlled synthesis of a desired polycation in this system: temperature and the utilization of different auxiliaries. In all reactions, elemental antimony and grey selenium were employed as main starting materials.

If SeCl_4_ is added to Sb and Se in [BMIm]Cl·*n*AlCl_3_, [Sb_10_Se_10_][AlCl_4_]_2_ can be accessed at room temperature [35]. However, if SeCl_4_ is omitted, either the *catena-*compound [Sb_2_Se_2_][AlCl_4_] or the cluster compound [Sb_13_Se_16_][AlCl_4_]_6_Al_2_Cl_7_ form depending on the reaction temperature [30,34]. Thereby, [Sb_13_Se_16_][AlCl_4_]_6_Al_2_Cl_7_ can be interpreted as intermediate during the decomposition of [Sb_2_Se_2_][AlCl_4_] into Sb_2_Se_3_. However, this thwarts the isolation of pure [Sb_13_Se_16_][AlCl_4_]_6_Al_2_Cl_7_ due to its narrow temperature range of existence.

The related *spiro*-heterocubanes [Sb_7_Se_8_Br_2_][Al*X*_4_]_3_, [Sb_13_Se_16_Br_2_][Al*X*_4_]_5_, and [Sb_7_Se_8_Br_2_][Sb_13_Se_16_Br_2_][AlBr_4_]_8_ are obtained from mixtures of [BMIm]Br with AlCl_3_, AlBr_3_, and/or NbCl_5_ at 160 °C [38]. If only AlBr_3_ is present, [Sb_7_Se_8_Br_2_][Sb_13_Se_16_Br_2_][AlBr_4_]_8_ precipitates exclusively. However, [Sb_13_Se_16_Br_2_][Al*X*_4_]_5_ crystalizes if AlCl_3_ is employed instead. Finally, by combining AlBr_3_ and NbCl_5_, [Sb_7_Se_8_Br_2_][Al*X*_4_]_3_ forms.

### 3.3. Bismuth-Tellurium Heteropolycations—Adjustments via Starting Materials

In a fashion similar to the aforementioned case of antimony and selenium, heteropolycations of bismuth and tellurium (and bromine) can be synthesized in Lewis-acidic ILs. In this system, however, the choice of starting materials seems to have the dominant influence on the obtained polycation.

Starting from elemental bismuth and tellurium, Kanatzidis et al. synthesized [Bi_2_Te_2_Br][AlCl_4_] and [(Bi_4_Te_4_Br_2_)(Al_2_Cl_5.46_Br_0.54_)]Cl_2_ in [EMIM]Br·4.8AlCl_3_ at 165 °C. Seemingly by changing the bismuth to tellurium ratio from 1:1 to 1:3, the latter compound was favored [33,45].

In chloride-free [BMIm]Br·4.1AlBr_3_, either the cube-shaped polycation [Bi_4_Te_4_]^4+^ or the realgar-like [Bi_6_Te_4_Br_2_]^4+^ forms at 100 °C depending on the employed starting materials [30]. If bismuth telluride and bismuth tribromide are reacted, [Bi_4_Te_4_][AlBr_4_]_4_ crystallizes as the sole polycation phase. Without addition of bismuth bromide, Bi_2_Te_3_ shows only low solubility in the employed IL and no formation of a solid product has been observed [30]. The reaction of bismuth, tellurium, and bismuth tribromide under the same conditions, however, leads to the crystallization of [Bi_6_Te_4_Br_2_][AlBr_4_]_4_.

In addition, [Bi_4_Te_4_][AlCl_4_]_4_ [105], which has initially been synthesized by Beck et al. in a NaCl·11AlCl_3_ melt at 130 °C, was synthesized by reacting bismuth telluride and bismuth trichloride in [BMIm]Cl·4.7AlCl_3_ at 100 °C. Thereby, the yield could be significantly improved compared to the original approach [30].

### 3.4. Manipulating the Stacking Order of Layered Compounds

Recently, we proved that the choice of starting materials can even influence the crystallized polytype of a layered compound, namely Cu_2_Bi_2_S_3_[AlCl_4_]_2_ [32]. In its structures, Bi_2_S_3_ molecules are connected by copper ions forming cationic layers that are separated by [AlCl_4_]^−^ tetrahedra. Upon dissolving Cu_3_Bi_2_S_3_Br_2_ [106] in [BMIm]Cl·3.6AlCl_3_ or [EMIm]Cl·3.6AlCl_3_ at 80 to 200 °C, approximately half of the precursor recrystallizes as Cu_2_Bi_2_S_3_[AlCl_4_]_2_ with a rhombohedral structure. In addition, small amounts of Bi_5_[AlCl_4_]_3_ (vide supra) and a hexagonal polytype are found. The compound is also accessible from CuCl, Bi_2_S_3_, and AlCl_3_ at 200 °C either by solvent-free reaction or by ionothermal synthesis in [BMIm]Cl·3.6AlCl_3_. Omitting the IL results in a larger portion of Bi_5_[AlCl_4_]_3_ while the rhombohedral polytype is again the main (but only microcrystalline) product. In contrast, reacting the binary starting materials in the IL strongly favors the crystallization of the hexagonal polytype. Substitution of CuCl with Cu_2_S and the corresponding amount of Bi_2_S_3_ with BiCl_3_ results in the crystallization of similar amounts of both polytypes from the IL.

### 3.5. Influence of Concentration and Ion Specification

Lewis-acidic ILs feature a remarkable solubility for metalloids and their halides, which extends to ternary compounds [29,107] with heavy transition metal elements like Bi_16_PdCl_22_ = [Pd@Bi_10_]Bi_6_Cl_22_ [108]. The dissolution of Bi_16_PdCl_22_ in the Lewis-acidic IL [BMIm]Br·4.1AlBr_3_ yields [Pd@Bi_10_](AlBr_4_)_2_(Al_2_Br_7_)_2_ or [Pd@Bi_10_](AlBr_4_)_4_ depending on temperature and/or AlBr_3_ to [Pd@Bi_10_]^4+^ ratio [107,109]. At 140 °C and with *n*(AlBr_3_):*n*([Pd@Bi_10_]^4+^) = 160:1, [Pd@Bi_10_][AlBr_4_]_2_[Al_2_Br_7_]_2_ crystallizes, while increasing the reaction temperature or bisecting the ratio yields [Pd@Bi_10_][AlBr_4_]_4_ instead.

The concentration- and/or temperature-driven preference for one of the two compounds can be rationalized with the anion specification in the IL or similar salt melts [5,100,110]. At high contents of the aluminum halide (i.e., strongly Lewis-acidic), adducts [Al_n_*X*_3n+1_]^−^ prevail. Their lower charge density and symmetry compared to [AlX_4_]^−^, however, strongly disfavors their incorporation into crystal structures. Increasing temperature breaks the adducts and increases the concentration of isolated tetrahedra [100]. A lower amount of dissolved [Pd@Bi_10_]^4+^ polycations leads apparently to the crystallization of tetrahedra pairs owing to the higher excess of pairs.

## 4. Conclusions

The scope of this review is to summarize and deduce decisive reactions parameters for controlled syntheses of polyions of heavy main-group elements in ILs. Without covering all possibilities, we want to demonstrate several (unexpected) pathways to tune reactions in ILs in order to synthesize new compounds. Aside from intuitive parameters such as temperature, concentration of starting materials, or their stoichiometry, syntheses in ILs offer additional ways to control the reaction products such as by the shape and charge-density of the IL cation. For instance, the choice of the starting material can influence the yielded polyion or its polymorph. Auxiliary compounds can be additionally introduced, which for instance subtly adjust the redox potential or influence the dimensionality of polyanions. In addition, general advantages of utilizing ILs are demonstrated such as easier handling of delicate volatile components, provision of dissolved species for further reactions, or substitution of toxic compounds. We would like to encourage readers to explore the abilities of ILs in synthesis because the variety of decisive reaction parameters promises an abundance of possible new compounds. We believe that many discussed reaction principles can be transferred to other classes of compounds, especially in main group-element chemistry.

## Figures and Tables

**Table 1:** Synopsis of used abbreviations of cations and anions of ILs in the review.

Abbreviation	Full Name
[EHIm]	1-ethylimidazolium
[EMIm]	1-ethyl-3-methylimidazolium
[PMIm]	1-propyl-3-methylimidazolium
[BMIm]	1-butyl-3-methylimidazolium
[HMIm]	1-hexyl-3-methylimidazolium
[DMIm]	1-dodecyl-3-methylimidazolium
[BMMIm]	1-butyl-2,3-dimethylimidazolium
[PMMIm]	1-propyl-2,3-dimethylimidazolium
[C_4_MPyr]	butyl-methylpyrrolidinium
[C_10_MPyr]	decyl-methylpyrrolidinium
[N(n-Bu)_3_Me]	tributylmethylammonium
[P_4444_]	tetrabutylphosphonium
[P_66614_]	trihexyl-tetradecylphosphonium
[P(Bz)(Ph)_3_]	benzyl(triphenyl)phosphonium
[CTf_3_]	tristriflylmethanide, tris(trifluoromethanesulfonyl)methanimide
[NTf_2_]	triflimide, bis(trifluoromethanesulfonyl)imide
[OTf]	triflate, trifluoromethanesulfonate

**Table 2:** Synthesis of carbonyl complexes of heavy main group elements. All reactions were performed at 130 °C in sealed ampules.

Carbonyl	Iodide	IL	Cluster Anion	Ref.
Mn_2_(CO)_10_	TeI_4_	[BMIm][OTf]	[(Te_2_)_3_{Mn(CO)_3_}_2_{Mn(CO)_4_}_3_]^−^	[80]
	PbI_2_	[BMIm][NTf_2_]	[(Pb_6_I_8_){Mn(CO)_5_}_6_]^2−^	[81]
Fe(CO)_5_	SnI_4_	[EMIm][NTf_2_]	[FeI(CO)_3_(SnI_3_)_2_]^−^	[82]
		[EHIm][NTf_2_]	[FeI(CO)_3_(SnI_3_)_2_]^−^	[82]
		[PMIm][NTf_2_]	[FeI(CO)_3_(SnI_3_)_2_]^−^	[82]
		[BMIm][NTf_2_]	[{Fe(CO)_3_}_4_Sn_6_I_10_]^2−^	[83]
		[BMIm][OTf]	[{Fe(CO)_3_}_4_Sn_6_I_10_]^2−^	[83]

**Table 3:** Influence of the amine content on the product distribution of ternary selenidostannates under otherwise identical reaction conditions. A dash indicates the absence of an identifiable product. The structural connectivity of the anionic part is indicated by 0D (cluster), 2D (layer), or 3D (framework). [K_4_(H_2_O)_3_][Ge_4_Se_10_] (56 mg), SnCl_4_∙5H_2_O (40 mg), the amine, and [BMMIm][BF_4_] (0.5 g) were annealed at 150 °C for two days and subsequently cooled to ambient temperature [92].

*V*/μL	2,6-Dimethylmorpholine = DMMP	ethylenediamine = en
0	–	–
10	–	0D-[BMMIm]_24_[Sn_36_Ge_24_Se_132_]
20	–	0D-[BMMIm]_24_[Sn_36_Ge_24_Se_132_]
30	0D-[BMMIm]_24_[Sn_36_Ge_24_Se_132_]	2D-[BMMIm]_2_[Ge_0.83_Sn_3.17_Se_9.06_]
50	0D-[BMMIm]_24_[Sn_36_Ge_24_Se_132_]	2D-[BMMIm]_2_[Ge_0.83_Sn_3.17_Se_9.06_]
100	0D-[BMMIm]_24_[Sn_36_Ge_24_Se_132_]	3D-[BMMIm]_8_[Sn_18_Se_40_]
	2D-[BMMIm]_2_[Ge_0.83_Sn_3.17_Se_9.06_]	
>200	microcrystalline SnSe_2_	–

**Table 4:** Impact of the amine content on the product distribution of binary selenidostannates under otherwise identical reaction conditions. Sn (1 mmol), Se (2.5 mmol), hydrazine hydrate, and [PMMIm]Cl (1 g) were annealed at 160 °C for 5 days and subsequently cooled to ambient temperature [90].

*n*(IL):*n*(hydrazine hydrate)	Product
(without amine)	3D-[PMMIm]_4_[Sn_9_Se_20.93_]-nanoparticles
4.9:1.0	3D-[PMMIm]_4_[Sn_9_Se_20.93_]
	2D-[PMMIm]_8_[Sn_17_Se_38_]
4.9:1.6	2D-[PMMIm]_8_[Sn_17_Se_38_]
2.0:15.0	2D-[PMMIm]_2_[Sn_3_Se_7_]
